# Artemisinin resistance threat in Central and West Africa needs holistic action

**DOI:** 10.4102/jphia.v17i1.1405

**Published:** 2026-01-16

**Authors:** Loick Pradel Kojom Foko, Amit Sharma

**Affiliations:** 1 Molecular Medicine Group, International Centre for Genetic Engineering and Biotechnology (ICGEB), New Delhi, India; 2 Department of Public Health, Center for Expertise and Research in Applied Biology (CEREBA), Douala, Cameroon

**Keywords:** malaria, drug resistance, Central Africa, West Africa, response

## Abstract

Artemisinin partial resistance (ART-R) has now emerged in the Horn, Eastern and Southern Africa. Mutations in the *Plasmodium falciparum kelch13* gene, strongly associated with ART-R, are increasingly reported in several Central and West Africa countries. Here, we opine that the emergence and spread of ART-R in Central and West Africa should not be overlooked given public health, clinical and economic consequences. Again, in addition to the recent funding cuts from the United States (US) government, some regions in these countries are affected by armed conflicts that undoubtedly will disrupt malaria control measures. Thus, measures should be proactively taken to prevent the emergence of ART-R or mitigate its spread in these two regions. We also propose strategies that could be valuable in implementing a near real-time surveillance and information system, will produce high-quality analysis, allow to draw malaria reality-reflecting conclusions, optimally enhance data use and define tailored control and elimination strategies.

The latest report from the World Health Organization (WHO) highlights a rise in the global malaria burden from 219 million cases in 2022 to 263 million in 2023.^1^ The precise cause of this increase remains unclear, but it is probably due to a complex action of several factors such as the emergence or re-emergence of originally zoonotic pathogens (e.g., coronavirus disease 2019 [COVID-19]), climate change, armed conflicts and decline in malaria control efforts. In addition, mosquito insecticide resistance, *Anopheles stephensi* invasion, low coverage of preventive strategies, the emergence of *Plasmodium falciparum* parasites escaping histidine-rich protein 2 gene (*pfhrp2*)-targeting rapid diagnostic tests, and the development of artemisinin partial resistance (ART-R) in Africa have likely all contributed.^1^

Artemisinin-based combination therapies (ACTs) are the backbone of the current therapeutic strategies to treat uncomplicated *P. falciparum* malaria in Africa, while intravenous artesunate is recommended for severe malaria.^2^ In Central and West African countries, ACTs such as artesunate + amodiaquine, artemether + lumefantrine and now dihydroartemisinin + piperaquine are commonly used against uncomplicated *P. falciparum* malaria. ART-R is defined as ‘a delay in the clearance of malaria parasites from the bloodstream following treatment with an ACT’ (https://www.who.int/news-room/questions-and-answers/item/artemisinin-resistance). Well-established in the Greater Mekong subregion (GMS), Southeast Asia, ART-R is now being reported in the Horn of Africa.^3^ Signals of ART-R have also been reported in other countries (e.g., Uganda, Rwanda).^1^

Since 2018, WHO proposed a targeted malaria response strategy referred to as ‘High Burden to High Impact (HBHI)’ in endemic countries. Now, the HBHI list is constituted totally by African countries, where there is a need for more aggressive malaria control efforts. Several Central and West African countries belong to HBHI countries (i.e., Nigeria, the Democratic Republic of the Congo [DRC], Niger, Angola, Burkina Faso, Benin, Guinea, Mali, Chad, Cameroon, Ghana and Côte d’Ivoire).^1^ These countries show contrasting epidemiological trajectories for malaria incidence and death cases during the last five years (2019–2023). In some countries in Central (e.g., Central African Republic) and West (e.g., Nigeria, Senegal) regions, an increase in both cases and deaths was observed during the time frame (Figure 1).

**FIGURE 1:**
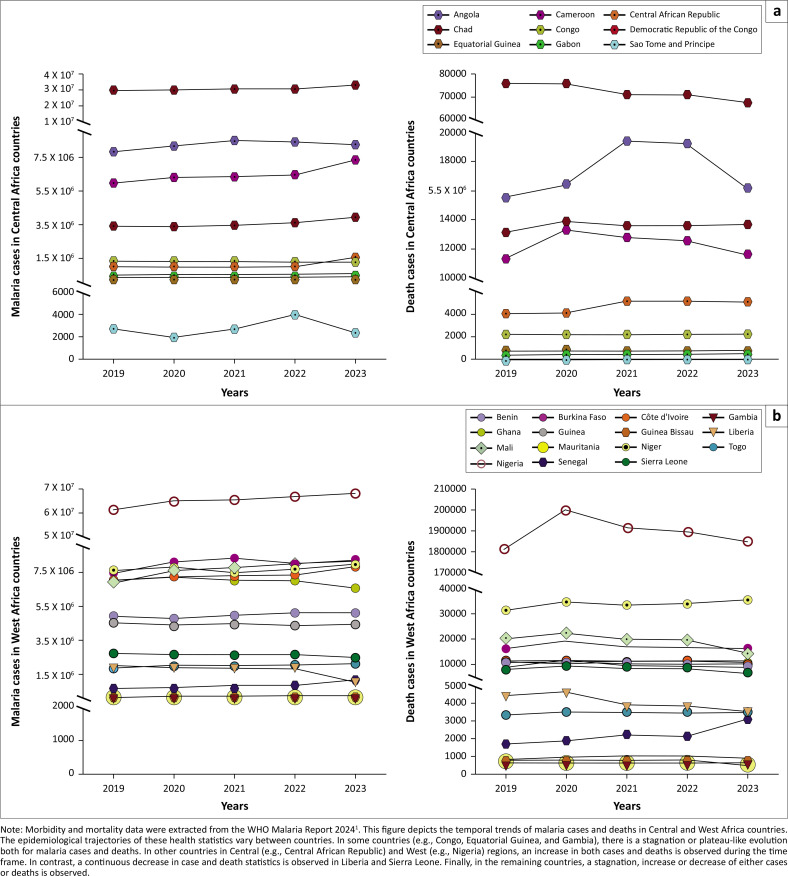
Malaria cases and deaths in Central (a) and West (b) Africa, 2019–2023.

Single nucleotide polymorphisms (SNPs) in the propeller domain of the *Kelch* 13 gene (*pfk*13) have been identified as major genetic drivers of ART resistance.^3,4^ Single nucleotide polymorphisms (SNPs) in the propeller domain of the *Kelch13* gene (*pfk13*) have been identified as major genetic drivers of ART resistance.^3^ To date, ~20 of these SNPs are either candidate or validated markers of ART resistance based on their strong *in vivo* and/or*in vitro* associations with delayed *P. falciparum* clearance (Figure 2).^5^ Some of these validated mutations, such as R539T and R561H are prevalent in Africa.^3^ Also, some studies outlined the role of *P. falciparum* genetic background and non-*pfk13* genes (e.g., coronin, or ferredoxin) in the acquisition of ART-R phenotype.^6^ Given the logistic, technical, financial and methodological challenges of clinical trials, typing of drug resistance *pfk13* SNPs is more frequently performed for ART-R resistance surveillance and research purposes. The scalability of next-generation sequencing (NGS) tools, such as Oxford Nanopore sequencing, allows for continuous, rapid and timely evaluation of drug resistance profiles.^7^

**FIGURE 2:**
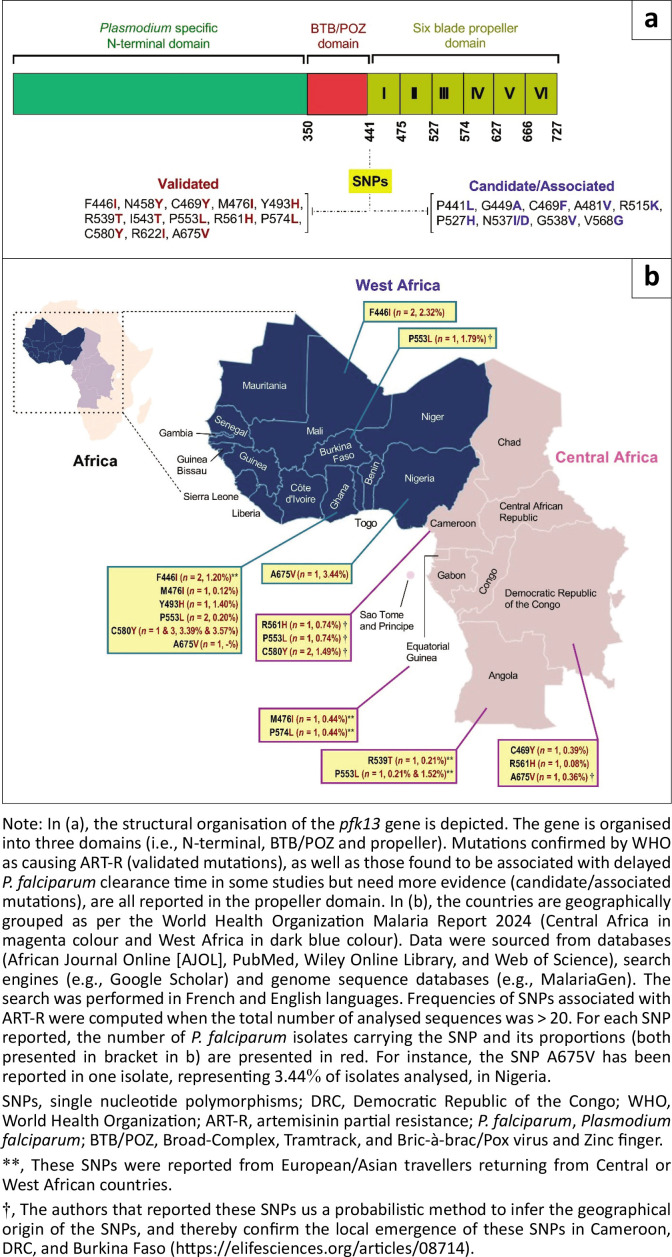
Schematic representation of the *pfkelch13* gene and single nucleotide polymorphisms (validated, candidate/associate) (a) and frequency of artemisinin resistance-related validated single nucleotide polymorphisms (b) in Central and West Africa.

Artemisinin partial resistance could pose a significant threat to malaria control efforts in Africa, particularly in Central and West regions of the continent. These two regions have so far remained unaffected by ART-R confirmation but are highly vulnerable because of high transmission rates and a substantial malaria burden (Figure 1). Recent cuts to infectious disease funding by the United States (US) government, particularly reductions in support from the President’s Malaria Initiative,^8^ are compounding these risks. These funding shortfalls could delay the procurement and distribution of ACTs, lead to interruptions in control interventions (e.g., long-lasting insecticide-treated nets, indoor residual spraying). This result in the scaling back of molecular surveillance programmes critical to the early detection of ART-R. Without adequate financial and technical support, health systems in these regions may struggle to detect and contain ART-R effectively, increasing the likelihood of its spread. Furthermore, funding cuts will hamper fight against malaria and could lead to dire outcomes in terms of public health and economic aspects.

Molecular signals arose from the eastern DRC (Central Africa) where ART-R *pfk13* mutant strains (R561H, P441L) were reported.^9^ Artemisinin partial resistance-related mutations have been reported at marginal rates in other countries (e.g., A675V [*n* = 1 isolate, 3.44%] in Nigeria, A675V [*n* = 1 isolate, 2.32%] in Mali, A675V [*n* = 1 isolate, 1.79%] in Burkina Faso, R561H [*n* = 1 isolate, 0.74%], P553L [*n* = 1 isolate, 0.74%] and C580Y [*n* = 2 isolates, 1.49%] in Cameroon) (Figure 2). Even though not adequately designed, a study reported reduced artemether + lumefantrine efficacy (i.e., treatment failure rates > 20%) in Gourcy and Nanoro, two cities in Burkina Faso where malaria transmission is stable and seasonal.^10^ However, these ACT efficacy data need further work in Central and West Africa countries. It should be noticed that Kelch13 mutations and ACT therapeutic efficacy data in these countries are mostly outdated, with *P. falciparum* isolated collected in 2019 for the most recent studies in these two regions (e.g., Benin, Gabon). Parasite surveillance is either limited or absent in other countries (e.g., Niger). This hence requires implementation of therapeutic efficacy and molecular studies for understanding the current status of ART-R in Central and West Africa countries.

Regions in Africa with armed conflicts will undoubtedly experience disruptions in malaria control measures. Also, areas where above mutants have been reported are geographically close to Rwanda and Uganda, two countries where ART-R is now evidenced.^1^ This suggests a possible transboundary human movement-driven expansion of these resistant strains. The world has become a veritable ‘planetary village’, and the corollary is an increased risk of globalisation of public health concerns such as ART-R. Other drivers of drug resistance, such as self-medication with antimalarial drugs, widespread use of artemisinin monotherapies, poor prescription practices, substandard and counterfeit drugs and partial ACT adherence, are prevalent in Africa. A recent study in Cameroon showed gaps between knowledge and prescription of ACTs among health caregivers. For instance, only 6.2% of patients receiving artemether + lumefantrine at the correct posology.^11^ In Ivory Coast, N’guessan et al. found that 26% and 7% of artemether + lumefantrine-containing drugs were underdosed and overdosed, respectively.^12^ In Nigeria, a study reported that artemisinin monotherapies were still available in the private market at rates of ~9.2% – 13.3% for artesunate and 9.5% for dihydroartemisinin.^13^ Thus, the risk of the *de novo* emergence, spread or importation of ART-R to Central and West Africa is high.^4^

Nonetheless, there are reasons for guarded optimism:

- ACT-based therapeutic options are still highly effective in most parts of the continent, even though some studies in Burkina Faso reported low ACT efficacy.^10,14^ Molecular epidemiology studies reported ART-R-associated *pfk13* SNPs at marginal frequencies (Figure 2), thereby suggesting a slower evolution of ART-R in these two African regions compared to the Greater Mekong subregion, Southeast Asia.^15,16^ In view of achieving WHO malaria control and elimination milestones by 2030, it is crucial to implement urgent and regional actions through a concerted collaborative framework for efficiently preventing ART-R in Central and West Africa. Such actions imply holistically addressing ART-R through both research and practical measures. It is of utmost importance for Central and West African Governments to be aware of the potential public health represented by ART-R, and thus, define a collaborative framework for coordinated regional actions and data sharing. The implementation of such regional actions requires boosting funding. Malaria endemic countries are still greatly dependent on external funding from international and national entities such as the US, or the European Commission.^1^
- Local actions tailored to each country should be developed, implemented or intensified to prevent ART-R appearance or halt its spread. For instance, combating self-medication with antimalarial drugs (i.e., artemisinin derivatives and partner drug), and concomitantly promoting better strategies for rational usage of ACTs is vital.^11^ There is a need to combat ART-R drivers, such as falsified and substandard drugs.^17^ It is essential to conduct studies focusing on evaluating the impact of self-medication and substandard or falsified drugs on artemisinin derivative and drug partner-related resistance polymorphisms.
- Molecular epidemiology and clinical studies should be conducted to appraise and document the burden of ART-R and *pfk13* SNPs. Areas with ART-R-related SNPs data or bordering areas should primarily target. Such action could be realised by utilising scalable and point-of-care NGS technologies. The recent COVID-19 pandemic highlighted the importance of setting up molecular detection centres for rapid detection and report of cases, mortality and viral variants. These centres could likely serve as a platform for molecular profiling of major pathogens including *P. falciparum* parasites.
- The ability to produce, report, extract and analyse ART-R-related data generated across the Central and West African regions should be enhanced. There are several independent public and private bodies generating malaria data (e.g., National Malaria Control, and non-government organisations), making such data fragmented and not easily exploitable.
- Awareness and engagement of populations are undoubtedly indispensable for the success of Government-driven local and regional malaria control and elimination actions. This has to be enhanced also.
- The role of scientists is fundamental for the success of political engagement and actions. Ongoing research, including gene-editing studies to evaluate SNP-related ART-R and fitness costs, is also essential.
